# “Obesity and Insulin Resistance” Is the Component of the Metabolic Syndrome Most Strongly Associated with Oxidative Stress

**DOI:** 10.3390/antiox11010079

**Published:** 2021-12-29

**Authors:** Grzegorz K. Jakubiak, Kamila Osadnik, Mateusz Lejawa, Tadeusz Osadnik, Marcin Goławski, Piotr Lewandowski, Natalia Pawlas

**Affiliations:** 1 Department of Pharmacology, Faculty of Medical Sciences in Zabrze, Medical University of Silesia, Jordana 38 Street, 41-808 Zabrze, Poland; kosadnik@sum.edu.pl (K.O.); mateusz.lejawa@sum.edu.pl (M.L.); tadeusz.osadnik@sum.edu.pl (T.O.); natalia.pawlas@sum.edu.pl (N.P.); 2 Department and Clinic of Internal Medicine, Angiology, and Physical Medicine, Faculty of Medical Sciences in Zabrze, Medical University of Silesia, Batorego 15 Street, 41-902 Bytom, Poland; 3 Student Research Group, Department of Pharmacology, Faculty of Medical Sciences in Zabrze, Medical University of Silesia, Jordana 38 Street, 41-808 Zabrze, Poland; martin.golawski@gmail.com (M.G.); lewandop@icloud.com (P.L.)

**Keywords:** oxidative stress, metabolic syndrome, obesity, insulin resistance, diabetes, hypertension, dyslipidemia

## Abstract

Metabolic syndrome (MS) is not a homogeneous entity, but this term refers to the coexistence of factors that increase the risk for the development of type 2 diabetes and cardiovascular disease. There are different versions of the criteria for the diagnosis of MS, which makes the population of patients diagnosed with MS heterogeneous. Research to date shows that MS is associated with oxidative stress (OS), but it is unclear which MS component is most strongly associated with OS. The purpose of the study was to investigate the relationship between the parameters of OS and the presence of individual elements of MS in young adults, as well as to identify the components of MS by means of principal components analysis (PCA) and to investigate how the parameters of OS correlate with the presence of individual components. The study included 724 young adults with or without a family history of coronary heart disease (population of the MAGNETIC study). Blood samples were taken from the participants of the study to determine peripheral blood counts, biochemical parameters, and selected parameters of OS. In addition, blood pressure and anthropometric parameters were measured. In subjects with MS, significantly lower activity of superoxide dismutase (SOD), copper- and zinc-containing SOD (CuZnSOD), and manganese-containing SOD (MnSOD) were found, along with significantly higher total antioxidant capacity (TAC) and significantly lower concentration of thiol groups per gram of protein (PSH). We identified three components of MS by means of PCA: “Obesity and insulin resistance”, “Dyslipidemia”, and “Blood pressure”, and showed the component “Obesity and insulin resistance” to have the strongest relationship with OS. In conclusion, we documented significant differences in some parameters of OS between young adults with and without MS. We showed that “Obesity and insulin resistance” is the most important component of MS in terms of relationship with OS.

## 1. Introduction

Cardiovascular diseases (CVDs) are a significant cause of mortality worldwide. Although the rate of death caused by CVD decreased in high-income countries between 1990 and 2017, it remained comparable in lower- and middle-income countries [1].

Metabolic syndrome (MS) is not a separate disease, but this term refers to the coexistence of risk factors for the development of type 2 diabetes (T2D) and CVD [2]. There is no doubt that obesity significantly increases the risk for the development of MS [3], but the relationship between obesity and MS is not fully understood. Among obese individuals, there are patients who show metabolic disorders (metabolically unhealthy obesity, MUO) as well as patients who do not (metabolically healthy obesity, MHO) [4]. In addition, there are also patients with MS and normal body weight [5].

There are different versions of the criteria for the diagnosis of MS. Each of them includes several elements, but not all are required to diagnose MS, which makes the population of patients with MS heterogeneous. MS definitions for children and adolescents have been developed by Weiss [6], Viner [7], De Ferranti [8], and Cook [9], and for adults by the World Health Organization [10], European Group for the Study of Insulin Resistance [11], Adult Treatment Panel III [12], and the International Diabetes Federation [13]. In a study conducted on a group of 1,205 overweight Caucasian children and adolescents, it was shown that, depending on the adopted criteria, the percentage of participants with diagnosed MS ranged from 6% to 39%, and only 2% met the diagnosis of MS according to all versions of criteria [14].

Mentioned criteria for the diagnosis of MS consider the following elements in different configurations: waist circumference (WC), waist-to-hip ratio (WHR), body mass index (BMI), blood pressure (BP), impaired fasting glucose (IFG), impaired glucose tolerance (IGT), insulin resistance (IR), total cholesterol blood concentration (TC), high-density lipoprotein cholesterol blood concentration (HDL-C), and triglyceride blood concentration (TG), but not low-density lipoprotein cholesterol (LDL-C). It has also been reported that MS is associated with an increased risk of other pathological conditions, such as liver steatosis [15], gallstone disease [16], infertility [17,18], and thrombosis [19].

Based on the studies conducted so far, there is no doubt that the presence of MS is associated with oxidative stress (OS) [20], but it is not clear which components make the most significant contribution to this relationship.

The purpose of this study is to investigate the association between the parameters of OS and the presence of MS and its components in young adults participating in the MAGNETIC study (metabolic and genetic profiling of young adults with and without a family history of premature coronary heart disease), as well as to identify the components of MS by means of principal components analysis (PCA) and to investigate how the parameters of OS correlate with the presence of individual components.

## 2. Materials and Methods

### 2.1. Study Population

**Figure 1:**
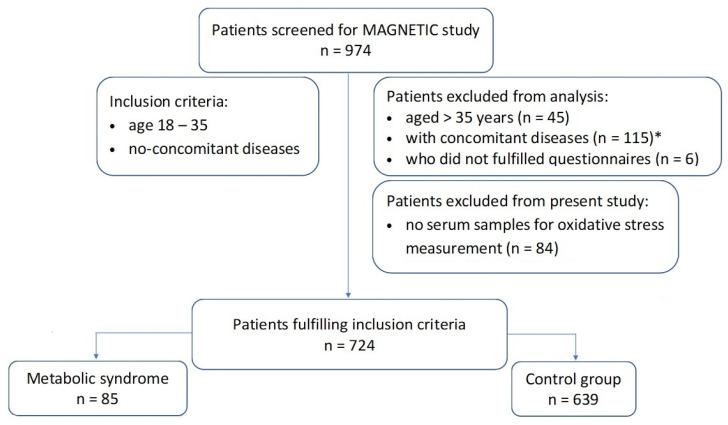
Study flowchart. Notes: * asthma or allergies (14); atopic skin disease (2); bipolar disorder or depression (3); cholelithiasis (1); chronic gastritis (1); coeliac disease (1); colitis (2); Crohn’s disease (1); diabetes mellitus (1); epilepsy (2); history of gastric ulcers (1); GERD (7); gout (2); Hashimoto’s disease and hypothyroidism (31); hypercholesterolemia treated with statins (1); hyperprolactinemia (1); hypertension (13); idiopathic purpura (1); irritable bowel syndrome (5); lactation (1); lactose intolerance (1); Marfan syndrome (1); migraines (3); nephrolithiasis (3); non-infectious hepatitis (1); polycystic ovarian disease (9); psoriasis (2); steatosis hepatitis (1); virial hepatitis (3).

The study group consisted of patients from the MAGNETIC study with complete clinical data enabling the diagnosis of MS in whom OS parameters were determined (n = 724). The study flowchart is shown in Figure 1. A detailed description of the MAGNETIC study design and research methodology has already been published previously [21]. Patient recruitment for the MAGNETIC study took place between June 2015 and December 2017. The main purpose of the MAGNETIC study was to analyze classical, genetic, and metabolic risk factors for coronary artery disease (CAD) in a population of healthy people aged 18–35 years with or without family history (FH) of premature CAD. A burdening FH was considered to be the occurrence of angiographically documented cases of CAD in a 1st degree relative before the age of 55 years (for men) or before the age of 65 years (for women). The reason why only young people without chronic diseases participated in the study is to ensure the homogeneity of the studied population and, in turn, obtain results demonstrating a relationship between the presence of MS and OS features with as few confounding factors as possible. The exclusion criteria were pregnancy, lactation, chronic diseases, and medication use (other than oral contraception). During the recruitment visit, patients completed a detailed questionnaire on sociodemographic data, possible medical history, and FH of various diseases, with particular emphasis on cardiological diseases and diabetes.

### 2.2. Blood Pressure and Anthropometric Measurements

BP was measured with a certified automatic apparatus. Weight (W), height (H), waist circumference (WC), and hip circumference (HC) were measured, and on the basis of the mentioned anthropometric measurements, the values of BMI and WHR were calculated for each participant according to the formulas:

BMI = (W [kg])/(H^2^ [m^2^]),

WHR = (WC [cm])/(HC [cm]).

### 2.3. Diagnosis of the Metabolic Syndrome

The diagnosis of MS was based on the definition of the joint position of the International Diabetes Federation, National Heart, Lung, and Blood Institute, American Heart Association, World Heart Federation, International Atherosclerosis Society, and International Association for the Study of Obesity. A prerequisite for the diagnosis of MS is that at least three of the following criteria are met: (1) central obesity defined as increased WC (cut-off values for male and female gender differ between populations and countries); (2) systolic BP (SBP) ≥ 130 mmHg or diastolic BP (DBP) ≥ 85 mmHg, or taking antihypertensive drugs by a patient with diagnosed arterial hypertension; (3) TG ≥ 150 mg/dL (1.7 mmol/L) or pharmacological treatment of this lipid disorder; (4) HDL-C < 40 mg/dL in men or <50 mg/dL in women, or pharmacological treatment for this lipid disorder; (5) fasting venous blood plasma glucose concentration ≥ 100 mg/dL, or pharmacological treatment of diagnosed carbohydrate metabolism disorders [22].

### 2.4. Laboratory Tests

Peripheral blood samples were collected from study participants between 7 and 9 a.m., approximately 8–10 h after the last meal. Laboratory tests were performed on Siemens BCS XP (Siemens Healthcare, Germany) and Cobas 6000 (Roche Diagnostics, Indianapolis, IN, USA). Concentrations of the following biochemical compounds were determined in each patient: glucose, blood lipid profile (TC, HDL-C, LDL-C, TG), lipoprotein(a) [Lp(a)], apolipoprotein A (apoA), apolipoprotein B (apoB), homocysteine, fibrinogen, creatinine, cystatin C, uric acid (UA), total protein (TP), albumin, bilirubin, thyrotropin (TSH), vitamin D, and high sensitivity C-reactive protein (hsCRP). In addition, the blood activity of the following enzymes was measured: alanine aminotransferase (ALT), aspartate aminotransferase (AST), gamma-glutamyl transpeptidase (GGT), alkaline phosphatase (ALP), and lactate dehydrogenase (LDH). Blood count and percentage of glycated hemoglobin (HbA1c) were also determined. The percentage of HDL-C was calculated according to the formula:

HDL-C [%] = (HDL-C/TC)∙100%.

The following parameters of OS were determined: total antioxidant capacity (TAC), total oxidative status (TOS), oxidative stress index (OSI), serum concentration of malondialdehyde (MDA), ceruloplasmin (CER), thiol group concentrations per gram of protein (PSH), lipid hydroperoxides (LPH), concentration of lipofuscin (LPS) in erythrocytes, the total activity of superoxide dismutase (SOD), as well as blood activity of isoenzymes such as copper- and zinc-containing superoxide dismutase (CuZnSOD) and manganese-containing superoxide dismutase (MnSOD). The methodology for determining the parameters of OS was described in detail by us in the recently published paper on the relationship between the parameters of OS and metabolic health in the participants of the MAGNETIC study [23].

### 2.5. Statistical Analysis

The distributions of continuous variables were compared with the Student’s *t*-test, which is plausible [24]. Dichotomous variables were compared with the Fisher test. In order to assess the spatial structure of the data and identify coexisting risk factors, factor analysis was performed. This method is exploratory and allows for the identification of hidden variables, which explain the maximum amount of variance in the dataset. This method consists of three steps. In the first stage, principal component analysis (PCA) is performed. The ingredients are then rotated to identify factors. In the last step, the factors are interpreted. The identified factors are independent of each other, and the degree of their correlation with the primary variables is determined by the so-called factor loadings. The higher the load, the greater the influence/share of a variable from the original dataset on a given factor. When naming the factors, the variables with the highest load are taken into account. The variables with a load above |0.4| were assumed to be significant in this analysis. The percentage of the variance of the original dataset explained by a given factor determines its significance.

## 3. Results

### 3.1. Characteristic of the Study Group

Table 1 shows a comparison of subgroups of study participants with and without diagnosed MS in terms of criteria such as age, attitude to smoking, education, financial situation, living conditions, physical activity, amount of sleep per day, place of residence, and results of anthropometric measurements. Patients with diagnosed MS were slightly older; there were more smokers among them, and they declared less physical activity compared to the group without diagnosed MS. Both groups did not differ significantly in terms of education, financial situation, living conditions, sleep duration, or place of residence. Among people with diagnosed MS, significantly higher values of WHR, BMI, SBP, and DBP were noted compared to people without MS.

Table 2 shows the differences between people with and without MS in terms of biochemical parameters, peripheral blood counts, and the percentage of HbA1c. The subgroups did not differ significantly in the concentration of Lp(a), bilirubin, TP, albumin, homocysteine, creatinine, and TSH. Among people with MS, significantly higher concentrations of TC, LDL-C, apoB, TG, glucose, fibrinogen, hsCRP, UA, and cystatin C were found, significantly higher number of white blood cells per unit of blood volume (WBC) and percentage of HbA1c, as well as significantly higher blood activities of ALT, AST, GGT, ALP, and LDH. On the other hand, among people with MS, significantly lower values of the concentration and percentage of HDL-C, apoA, and vitamin D levels were found.

### 3.2. Oxidative Stress Parameters

Table 3 presents the differences in the parameters of OS between people with and without diagnosed MS. There were no significant differences in the values of such parameters as CER, TOS, OSI, LPH, LPS, and MDA. Among people with MS, significantly lower activities of SOD, CuZnSOD, and MnSOD were found, as well as significantly higher value of TAC and significantly lower concentration of thiol groups per gram of protein.

### 3.3. The Results of PCA Analysis

PCA identified three components of MS that together accounted for 68% of the observed variance. The factor loadings for the rotated components (RC) are presented in Table 4. The first component (“Obesity and insulin resistance”) was characterized by high BMI (0.82), WC (0.85), glucose (0.59), TG (0.45), HbA1c (0.48), and low HDL-C (−0.73). The second component (“Dyslipidemia”) was characterized by high levels of TC (0.98), LDL-C (0.89), and TG (0.48). The third component (“Blood pressure”) was characterized by high SBP (0.88) and high DBP (0.89).

### 3.4. Correlations of the Main Components and Parameters of Oxidative Stress

**Figure 2:**
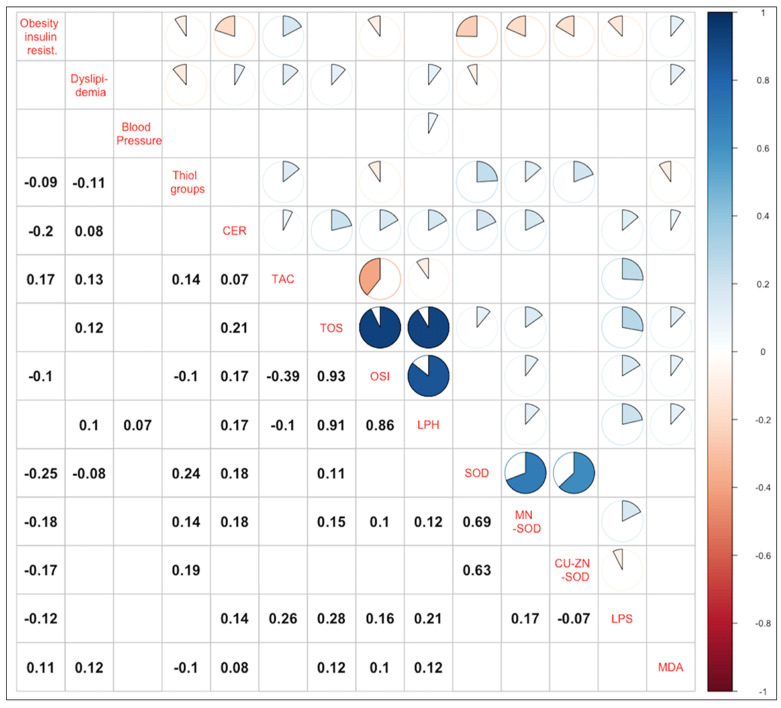
Correlation between main components and OS parameters. In the upper part of the plot, correlation coefficients between variables are presented in the form of a pie-chart. In the lower part of the plot, Spearman correlation coefficients are given. For clarity only, correlation coefficients with *p*-value < 0.05 are presented. CER—ceruloplasmin; TAC—total antioxidant capacity; TOS—total oxidative status; OSI—oxidative stress index; LPH—lipid hydroperoxides; SOD—superoxide dismutase; MnSOD—manganese-containing superoxide dismutase; CuZnSOD—copper- and zinc-containing superoxide dismutase; LPS—lipofuscin; MDA—malondialdehyde.

Figure 2 shows the Pearson correlation coefficients between the parameters of OS and main MS components. The component “Obesity and insulin resistance” (RC1) showed the strongest correlations with the parameters of OS, mainly with the concentration of CER (r = −0.24; *p* < 0.0001) and the activity of SOD (r = −0.23; *p* < 0.0001) in plasma. Weak but statistically significant correlations were also found between the RC1 and LPS concentration, as well as the activity of MnSOD and CuZnSOD in plasma, and between the RC1 and TAC. RC2 (“Dyslipidemia”) turned out to be more weakly correlated with the parameters of OS than RC1, and its strongest statistically significant correlation was the positive correlation with TAC. RC3 variable was poorly correlated with parameters of OS. Detailed data on the r-value for significant statistical correlations are presented in Figure 2. Main contributions of the variables to the principal components are shown in Figure S1.

## 4. Discussion

The most important achievement of our study is demonstration that the MS is significantly related to the parameters of OS, as well as evidence that the most important contribution to the presence of OS in the course of MS is made by the component associated with obesity and IR. We found significant differences between patients with MS and patients without MS in such parameters as SOD, MnSOD, CuZnSOD, TAC, and PSH, with no significant differences in such parameters as CER, TOS, OSI, LPH, LPS, and MDA.

We recently published the results of the analysis of data also from the participants of the MAGNETIC study, in which we investigated the differences in the parameters of OS between individuals with the MUO and MHO phenotypes, as well as metabolically healthy normal weight (MHNW) individuals. Despite similar research issues, the methodology of both these studies differs significantly. In the previous study, the compared groups were distinguished based on body weight and the presence or absence of metabolic disorders in the course of obesity [23]. In the current study, we analyzed which components of MS are associated with OS and compared differences between patients with and without MS, regardless of their body weight.

### 4.1. Obesity and Insulin Resistance and Oxidative Stress

The relationship between diabetes and OS is bidirectional. Hyperglycemia increases the markers of inflammation and OS, which leads to dysfunction of the cardiovascular system. OS and the chronic inflammatory process contribute to the development of IR and insulin secretion dysfunction, leading to the development of prediabetes and diabetes [25,26,27]. Mitochondrial OS was also shown to take part in the pathogenesis of IR [28], and on the other hand, hyperglycemia induces the mitochondrial production of pro-oxidative factors [29].

It was shown in a ten-year observational study that the parameters of inflammation, endothelial dysfunction, and OS are positively correlated with the risk of the development of T2D. For parameters of OS (F2-isoprostanes and oxidized LDL), the relationship was weaker after taking into account BMI values [30]. In obese children aged 3 to 6 years, selected parameters of OS, such as MDA, SOD, and 3-nitrotyrosine (3-NT), are positively correlated with BMI [31].

Interventions leading to weight loss in obese subjects may be associated with reduction of OS, although the data in the literature are not unambiguous. Eight weeks of caloric restriction was shown to be associated with a decrease in blood myeloperoxidase activity and an increase in SOD activity [32]. It has also been documented that in patients with morbid obesity, OS is reduced after bariatric surgery [33,34]. On the other hand, a study involving obese young women treated for infertility lifestyle modification did not cause any significant change in OS assessed by fasting serum-free thiols concentration [35].

Genetic factors may play a role in the relationship between OS and obesity and related diseases. Interestingly, according to the research conducted by Lewandowski et al., the genotype C/T of a single nucleotide polymorphism (SNP) rs4880 of the *SOD2* gene, encoding MnSOD, was present in 90% of obese individuals in the population studied by this group, what may indicate that this is a hereditary factor, playing a role in the development of obesity [36]. Inherited deficiency of catalase (CAT) is associated with an increased risk of carbohydrate metabolism disorders [37,38] and liver steatosis [39]. Moreover, OS related to excessive H_2_O_2_ was shown to be associated with increased body fat mass via both adipogenesis and lipogenesis, which is related to overexpression of nicotinamide adenine dinucleotide phosphate oxidase 4 (NOX4) and decreased expression of adenosine monophosphate-activated protein kinase (AMPK) in adipocytes [40].

In our results, blood activity of total SOD, as well as MnSOD and CuZnSOD, were negatively correlated with “Obesity and insulin resistance”. Similar results were presented by Jia et al. SOD blood activity was shown to negatively correlate with values of BMI and homeostatic model assessment for insulin resistance (HOMA-IR). In this study, normal weight, overweight, and obese males aged 22–58 participated [41]. MnSOD activity is significantly decreased in obese children aged 8-16 years old when compared to normal-weight children [42]. On the other hand, Fuentes-Venado et al. documented recently that SOD activity is significantly increased in obese children (3–6 years old) when compared to normal-weight children [31]. According to Dzięgielewska-Gęsiak et al., there is no significant difference in CuZnSOD activity in erythrocytes between healthy elderly subjects with and without IR [43], but CuZnSOD activity in erythrocytes was shown to be significantly decreased in elderly individuals with prediabetes [44].

According to our results, CER concentration is negatively correlated with “Obesity and insulin resistance”, but there is no significant difference in CER concentration between patients with and without MS. Safavi et al. did not notice any significant difference in CER concentration between obese and normal-weight subjects [45]. In obese adolescents, CER was documented to be positively correlated with IR assessed by HOMA-IR [46]. Kim et al. presented that CER concentration is increased in subjects with MS, prediabetes, and diabetes [47]. In postmenopausal women, CER is significantly elevated in subjects with prediabetes, but not in individuals with diabetes [48].

### 4.2. Dyslipidemia and Oxidative Stress

Oxidative modification of LDL particles plays an essential role in the pathogenesis of atherosclerosis [49]. Post-prandial lipemia was shown to be associated with increased OS in patients with T2D as well as in healthy subjects [50]. On the other hand, excess OS causes impaired insulin action, which leads to increased production and decreased clearance of very-low-density lipoproteins (VLDL) [51]. Interestingly, lipoprotein apheresis in patients with familial hypercholesterolemia is associated with significant improvement in OS parameters [52].

According to our results, TAC is the OS parameter that shows the strongest correlation with “Dyslipidemia”. In another study, it was shown that in young adults (30 ± 5 years) with sickle cell anemia TC, LDL-C, and TG significantly correlated with TAC [53]. On the other hand, in a study involving patients with vitiligo and healthy volunteers, it was found that the TAC value was significantly lower in the study group, while in the same group, the mean values of TC, LDL-C, and HDL-C were significantly higher, with no significant difference in TG concentration [54]. The participants in this study were older than in our population. In addition, the influence of vitiligo itself, which may be important here, must be taken into account.

### 4.3. Arterial Hypertension and Oxidative Stress

Simic et al. showed that in patients with all stages of arterial hypertension, increased oxidative protein damage can be found. The activity of extracellular antioxidant enzymes (such as SOD, glutathione peroxidase, and CAT) was significantly reduced in patients with moderate or severe hypertension compared to the control group [55]. Maciejczyk et al. showed that in children with hypertension, activities of SOD, CAT, and peroxidase, as well as the level of advanced glycation end products, MDA concentration, and nitrosative stress markers (peroxynitrite and nitrotyrosine), were significantly higher in non-stimulated and stimulated whole saliva and erythrocytes [56].

The concentration of 8-iso-prostaglandin F2α (8-iso-PGF2α) in the blood is one of the markers of OS and shows a direct vasoconstrictive effect mediated by thromboxane A2 receptors, which may indicate the contribution of OS to the pathogenesis of arterial hypertension [57,58]. In the course of hypertension, OS contributes to the activation of matrix metalloproteinases (MMPs), leading to vasoconstriction, endothelial dysfunction, promotion of cell migration, and vascular remodeling [59].

### 4.4. Metabolic Syndrome and Oxidative Stress

Janczura et al., in a study conducted on a group of police officers, showed that OS, assessed by the measurement of blood 8-iso-PGF2α concentration, depends on the number of components of MS [60]. In a study conducted by Korkmaz et al., in which only people with BMI below 25 kg/m^2^ participated, it was shown that the levels of advanced protein oxidation products, TAC, and the prooxidant-antioxidant balance are significantly higher in people with MS compared to people in the control group [61]. In our study, the TAC value was also significantly higher in people with MS, although our study included subjects with normal body weight as well as overweight and obese people, and the mean BMI and WHR values were significantly higher in people with MS.

Research conducted so far suggests that individual components of MS are not independent, but at least in part contribute to a common pathogenetic process, and OS is suspected to be its important element. Ma et al. showed that TG correlates with IR and beta-cell dysfunction even in people who do not meet the criteria for diagnosis of carbohydrate metabolism disorders (diabetes and prediabetes). In addition, the same study found that SOD activity negatively correlates with TG [62].

In our results, SOD activity was significantly lower among people with MS. Partially different results were presented by Vichaibun et al. According to this publication, TAC significantly decreases, and SOD activity significantly increases in people with hyperglycemia as well as hyperglycemia and dyslipidemia compared to the control group, without a significant difference between people with hyperglycemia and hyperglycemia coexisting with dyslipidemia [63]. Cardona et al. found no significant difference between SOD activity between patients with and without MS both at baseline and after fat overload [64]. In obese children and adolescents, no significant difference in SOD activity was found between individuals with MS and individuals meeting one or two criteria of MS (pre-MS) [65].

CuZnSOD activity was significantly lower in subjects with MS in our study. A similar result was obtained in a study conducted by Darroudi et al., in which 9,154 individuals with no diagnosed CVD participated [66]. According to Vávrová et al., patients with MS have significantly higher CuZnSOD blood activity, but what is worth mentioning, in this study, the activity of CuZnSOD was measured in erythrocytes [67].

Thiol group concentration is significantly lower in patients with MS, according to our results. It has already been shown that the concentration of thiol groups is significantly lower in patients with MS [68] as well as in patients with hypercholesterolemia [69].

### 4.5. Principal Component Analysis in Research on the Metabolic Syndrome

The PCA method has already been used in research on MS. Loos et al. used this method to identify chromosomal regions that may contain genes of products that might play a role in the development of MS [70]. Chang et al. showed that the equal-weighted average (EWA) could be used as a single parameter to identify individuals at risk of MS in clinical practice [71]. EWA is a function of BMI and age, according to the following formula:

EWA = 0.28∙BMI + 0.05∙age.

PCA was used to develop a formula to calculate a metabolic syndrome severity score, which was shown to be useful in the assessment of cardiometabolic risk factors [72]. A number of papers on the importance of dietary patterns that were identified using PCA in the context of MS have also been published [73,74,75].

According to the best of our knowledge, there is no article in the publicly available literature on the association between OS parameters and MS in which PCA was used.

### 4.6. Strengths and Limitations of the Study

As presented, on the basis of the research to date, the relationship between OS and MS and its individual components is beyond doubt, but the results of the studies conducted so far did not answer the question of which the MS component is the most important for the presence of OS. The important achievement of our study is the use of the PCA method in statistical analysis, which allowed us to show that the greatest contribution to the increased OS in people with MS is associated with the component of “Obesity and insulin resistance”. The study involved young adults without chronic diseases, not using medications (except oral contraception), and without a significant medical history. Moreover, it should be noticed that the group of subjects participating in our study is relatively homogenous.

Our study also has some limitations. The presented study is a case-control study that allows us to recognize the presence of certain correlations without the discovery of a cause-and-effect relationship. It should be noted that our work in the field of OS assessment did not investigate markers of protein and nucleic acid damage under the influence of reactive oxygen species as well as OS in red blood cells. A limitation of our study is also the lack of insulin determination and IR assessment (by HOMA-IR) in the studied population.

## 5. Conclusions

The relationship between MS and its components with OS is a widely discussed issue. However, the results of the studies conducted so far did not answer the question of which component of MS is the most important in terms of its contribution to the phenomenon of OS. The most important achievement in the presented study was the identification of the three main components of MS using PCA analysis and demonstration that the “Obesity and insulin resistance” component shows the strongest correlation with OS. Moreover, we showed significant differences in the parameters of OS between young adults with and without MS. We have shown that young adults with MS are characterized by the significantly lower activity of SOD, both total, and MnSOD as well as CuZnSOD isoenzymes. We also showed that young adults with MS are characterized by the significantly higher TAC value and the significantly lower concentration of thiol groups per gram of protein.

MS is a significant problem in modern medicine. A better understanding of its pathogenesis, including the relationship between MS and OS, may become the basis for further research to improve the quality of diagnosis and treatment.

## Figures and Tables

**Table 1:** The comparison of clinical and sociodemographic findings between young adult patients with and without MS.

Variable	Total Sample	Metabolic Syndrome		*p*-Value ^†^
		Yes	No	
N (%)	724 (100)	85 (11.7)	639 (88.3)	
Age [years]	27.9 ± 4.51	30.91 ± 3.34	27.51 ± 4.49	<0.0001
Current vs. never and former smoking (%)	157 (21.9)	26 (31.0)	131 (20.7)	0.04
Higher education vs. other types of education (%)	402 (55.5)	46 (54.1)	356 (55.7)	0.25
Financial situation above average vs. at or below average (%)	178 (24.6)	17 (20.0)	161 (25.2)	0.16
Living conditions average or modest vs. good or very good (%)	313 (43.3)	41 (48.2)	272 (42.6)	0.55
Physical activity low or average vs. high (%)	522 (72.1)	74 (87.1)	448 (70.1)	0.0002
Daily hours of sleep 6 or less vs. above 6 (%)	254 (35.2)	35 (41.2)	219 (34.4)	0.4
Place of residence: village or city below 20,000 residents vs. city above 20,000 residents (%)	253 (34.9)	33 (38.8)	220 (34.4)	0.83
**Physical examination**				
BMI [kg/m^2^]	24.31 ± 4.40	30.91 ± 4.54	23.43 ± 3.56	<0.0001
Waist men [cm] (n = 404)	88.01 ± 10.82	101.76 ± 11.81	85.84 ± 8.9	<0.0001
Waist women [cm] (n = 309)	74.22 ± 11.4	97.85 ± 11.12	72.05 ± 8.66	<0.0001
WHR	0.83 ± 0.09	0.93 ± 0.08	0.83 ± 0.09	<0.0001
SBP [mmHg]	126.51 ± 13.97	138.64 ± 14.96	124.9 ± 13.01	<0.0001
DBP [mmHg]	78.59 ± 10.52	87.53 ± 11.43	77.4 ± 9.81	<0.0001

^†^ Welch’s two-sample *t*-test for numerical variables. Pearson’s Chi-squared test for categorical variables.

**Table 2:** The comparison of laboratory findings between young adult patients with and without MS.

Variable	Total Sample	Metabolic Syndrome		*p*-Value ^†^
		Yes	No	
N (%)	724 (100)	85 (11.7)	639 (88.3)	
**Lipids**				
Cholesterol [mmol/L]	4.94 ± 1.02	5.62 ± 1.06	4.84 ± 0.98	<0.0001
LDL-C [mmol/L]	2.96 ± 0.94	3.66 ± 0.89	2.87 ± 0.9	<0.0001
Apolipoprotein B [g/L]	0.93 ± 0.43	1.18 ± 0.28	0.9 ± 0.43	<0.0001
HDL-C [mmol/L]	1.58 ± 0.44	1.13 ± 0.3	1.64 ± 0.42	<0.0001
HDL-C [%]	33.33 ± 10.78	20.81 ± 6.66	34.99 ± 10.11	<0.0001
Apolipoprotein A [g/L]	1.62 ± 0.32	1.45 ± 0.29	1.64 ± 0.31	<0.0001
Triglycerides [mmol/L]	1.18 ± 1.02	2.65 ± 2.07	0.99 ± 0.55	<0.0001
Lp(a) [nmol/L]	41.86 ± 63.28	49.14 ± 64.16	40.89 ± 63.15	0.27
**Liver cells**				
AST [IU/L]	23.59 ± 21.01	27.85 ± 14.35	23.02 ± 21.69	0.008
ALT [IU/L]	25.45 ± 23.6	41.22 ± 28.03	23.36 ± 22.14	<0.0001
GGT [IU/L]	26.55 ± 29.82	55.4 ± 57.35	22.71 ± 21.19	<0.0001
LDH [IU/L]	173.91 ± 38.45	188.33 ± 27.03	171.99 ± 39.35	<0.0001
Bilirubin [μmol/L]	11.43 ± 6.35	10.71 ± 6	11.53 ± 6.39	0.24
ALP [IU/L]	64.65 ± 19.58	73.06 ± 16.98	63.54 ± 19.64	<0.0001
**Glucose metabolism**				
Glucose [mmol/L]	5.01 ± 0.45	5.45 ± 0.54	4.95 ± 0.41	<0.0001
HbA1c [%]	4.99 ± 0.26	5.16 ± 0.26	4.95 ± 0.26	<0.0001
Total protein [g/L]	75.24 ± 5.14	74.89 ± 3.7	75.29 ± 5.3	0.38
**Remaining lab test results**				
WBC [×10^9^ /L]	5.85 ± 1.49	6.58 ± 1.63	5.75 ± 1.44	<0.0001
Albumin [g/L]	47.61 ± 3.32	47.18 ± 3.25	47.66 ± 3.32	0.20
Homocysteine [μmol/L]	11.69 ± 4.43	12.96 ± 7.54	11.52 ± 3.82	0.09
Fibrinogen [mg/dL]	275.08 ± 64.65	305.77 ± 72.17	271.01 ± 62.52	0.0001
hsCRP [mg/dL]	1.77 ± 2.74	2.47 ± 2.65	1.68 ± 2.74	0.01
Uric acid [μmol/L]	310.11 ± 75.8	368.95 ± 67.52	302.29 ± 73.4	<0.0001
Cystatin C [mg/dL]	0.81 ± 0.11	0.84 ± 0.12	0.8 ± 0.1	0.01
Creatinine [μmol/L]	78.1 ± 13.78	80.08 ± 13.95	77.83 ± 13.75	0.16
TSH [mU/L]	2.06 ± 1.11	2.23 ± 1.61	2.04 ± 1.03	0.29
Vitamin D [ng/mL]	22.51 ± 11.02	20.09 ± 11.06	22.83 ± 10.98	0.03

^†^ Welch’s two-sample *t*-test.

**Table 3:** The comparison of OS parameters between young adult patients with and without MS.

Variable	Total Sample	Metabolic Syndrome		*p*-Value ^†^
		Yes	No	
N (%)	724 (100)	85 (11.7)	639 (88.3)	
PSH [μmol/g protein]	4.6 ± 0.77	4.4 ± 0.88	4.62 ± 0.77	0.03
CER [mg/dL]	44.04 ± 14.49	44.03 ± 14.47	44.04 ± 14.5	0.99
TAC [mmol/L]	1.07 ± 0.15	1.11 ± 0.17	1.06 ± 0.15	0.01
TOS [μmol/L]	11.91 ± 34.05	13.01 ± 37.6	11.76 ± 33.58	0.77
OSI [%]	1.13 ± 3.16	1.16 ± 3.12	1.13 ± 3.17	0.94
LPH [μmol/L]	5.9 ± 17.66	7.07 ± 21.71	5.74 ± 17.06	0.59
SOD [NU/mL]	20.72 ± 2.49	19.45 ± 2.43	20.89 ± 2.45	<0.0001
MnSOD [NU/mL]	11.01 ± 1.84	10.57 ± 1.85	11.07 ± 1.83	0.02
CuZnSOD [NU/mL]	9.71 ± 1.93	8.86 ± 1.8	9.82 ± 1.93	<0.0001
LPS [RU/L]	272.96 ± 138.26	255.93 ± 172.18	275.23 ± 133.11	0.32
MDA [μmol/L]	2.19 ± 2.4	2.3 ± 2.41	2.17 ± 2.41	0.64

^†^ Welch’s two-sample *t*-test; TAC—total antioxidant capacity; TOS—total oxidative status; OSI—oxidative stress index; LPH—lipid hydroperoxides; SOD—superoxide dismutase; MnSOD—manganese-containing superoxide dismutase; CuZnSOD—copper- and zinc-containing superoxide dismutase; MDA—malondialdehyde.

**Table 4:** Variable loadings and percent of variance explained by each principal component.

Parameters		Factor Loadings	
	RC1 “Obesity and Insulin Resistance”	RC2 “Dyslipidemia”	RC3 “Blood Pressure”
BMI [kg/m^2^]	0.82		
WC [cm]	0.85		
SBP [mmHg]			0.88
DBP [mmHg]			0.89
TC [mmol/L]		0.98	
LDL [mmol/L]		0.89	
HDL [mmol/L]	−0.73		
TG [mmol/L]	0.45	0.48	
Glucose [mmol/L]	0.59		
HbA1c [%]	0.48		
Variance explained	28%	22%	18%
Cumulative variance explained	28%	50%	68%

Factor loadings of >|0.40| are shown in the table. Total variance in dietary variables explained by three patterns is 68%.

## Data Availability

The data presented in this study are available on request from the corresponding author. The data are not publicly available due to the planned preparation of subsequent publications based on the collected dataset (data may be publicly available after the end of the project, currently only upon reasonable request).

## Supplementary Materials

The following are available online at https://www.mdpi.com/article/10.3390/antiox11010079/s1, Figure S1: Main contributions of the variable to the principal components.

## Abbreviations

3-NT	3-nitrotyrosine
8-iso-PGF2α	8-iso-prostaglandin F2α
ALP	alkaline phosphatase
ALT	alanine aminotransferase
AMPK	adenosine monophosphate-activated protein kinase
AST	aspartate aminotransferase
apoA	apolipoprotein A
apoB	apolipoprotein B
BMI	body mass index
BP	blood pressure
CAD	coronary artery disease
CAT	catalase
CER	ceruloplasmin
CuZnSOD	copper- and zinc-containing superoxide dismutase
CVD	cardiovascular disease
DBP	diastolic blood pressure
EWA	equal-weighted average
FH	family history
GGT	gamma-glutamyl transpeptidase
H	height
HbA1c	glycated hemoglobin
HC	hip circumference
HDL	high-density lipoprotein
HDL-C	high-density lipoprotein cholesterol blood concentration
HOMA-IR	homeostatic model assessment for insulin resistance
hsCRP	high-sensitive C-reactive protein
IFG	impaired fasting glucose
IGT	impaired glucose tolerance
IR	insulin resistance
LDH	lactate dehydrogenase
LDL	low-density lipoprotein
LDL-C	low-density lipoprotein cholesterol blood concentration
Lp(a)	lipoprotein(a)
LPH	lipid hydroperoxides
LPS	lipofuscin
MAGNETIC	Metabolic and genetic profiling of young adults with and without a family history of premature coronary heart disease
MDA	malondialdehyde
MHNW	metabolically healthy normal weight
MHO	metabolically healthy obese
MMP	matrix metalloproteinase
MnSOD	manganese-containing superoxide dismutase
MS	metabolic syndrome
MUO	metabolically unhealthy obese
NOX4	nicotinamide adenine dinucleotide phosphate oxidase 4
OS	oxidative stress
OSI	oxidative stress index
PCA	principal component analysis
PSH	thiol groups concentration per gram protein
RC	rotated component
SBP	systolic blood pressure
SNP	single nucleotide polymorphisms
SOD	superoxide dismutase
T2D	type 2 diabetes
TAC	total antioxidant capacity
TC	total cholesterol blood concentration
TG	triglyceride blood concentration
TOS	total oxidative status
TP	total protein
TSH	thyrotropin
UA	uric acid
VLDL	very-low-density lipoprotein
W	weight
WBC	number of white blood cells per unit of blood volume
WC	waist circumference
WHR	waist-to-hip ratio
