# Supplementary material for: “Obesity and Insulin Resistance” Is the Component of the Metabolic Syndrome Most Strongly Associated with Oxidative Stress
Source: Antioxidants (Basel). 2021 Dec 29;11(1):79. doi: 10.3390/antiox11010079 (PMC8773170; doi:10.3390/antiox11010079)
Supplement: Supplementary file 1 [file antioxidants-11-00079-s001.zip › antioxidants-1502160-supplementary.pdf]

Figure S1. Main contributions of the variables to the principal components. (**A**) Component 1 ("Obesity and insulin resistance") vs. Component 2 ("Dyslipidemia"); (**B**) Component 2 ("Dyslipidemia") vs. Component 3 ("Blood pressure"); (**C**) Component 1 ("Obesity and insulin resistance") vs. Component 3 ("Blood pressure"). Patients with MS are presented as blue triangles whilst patients without MS are presented as yellow circles.

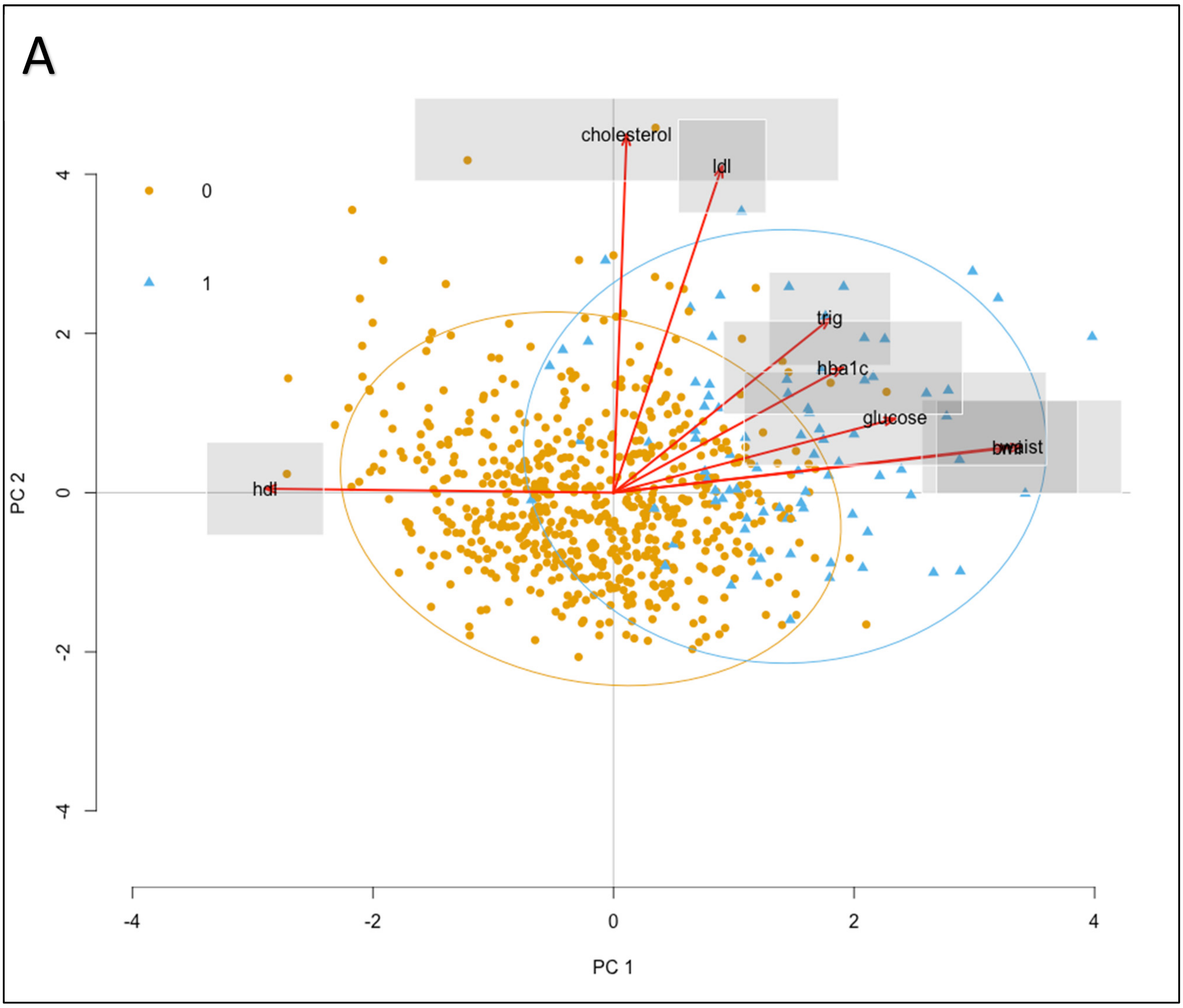

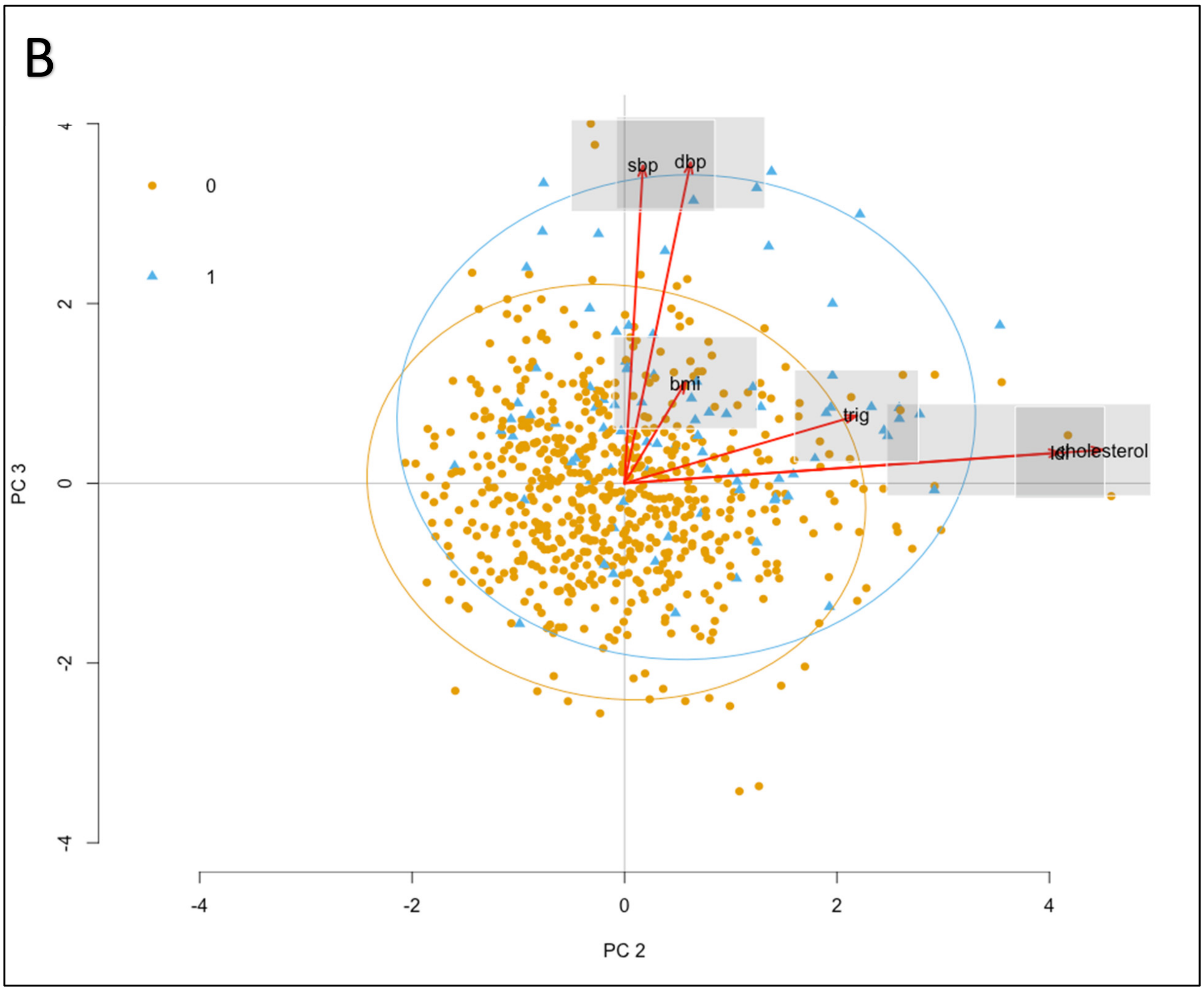
B
0
1
sbp
dbp
bmi
trig
ldl
cholesterol
PC 3
PC 2
-4
-2
0
2
4

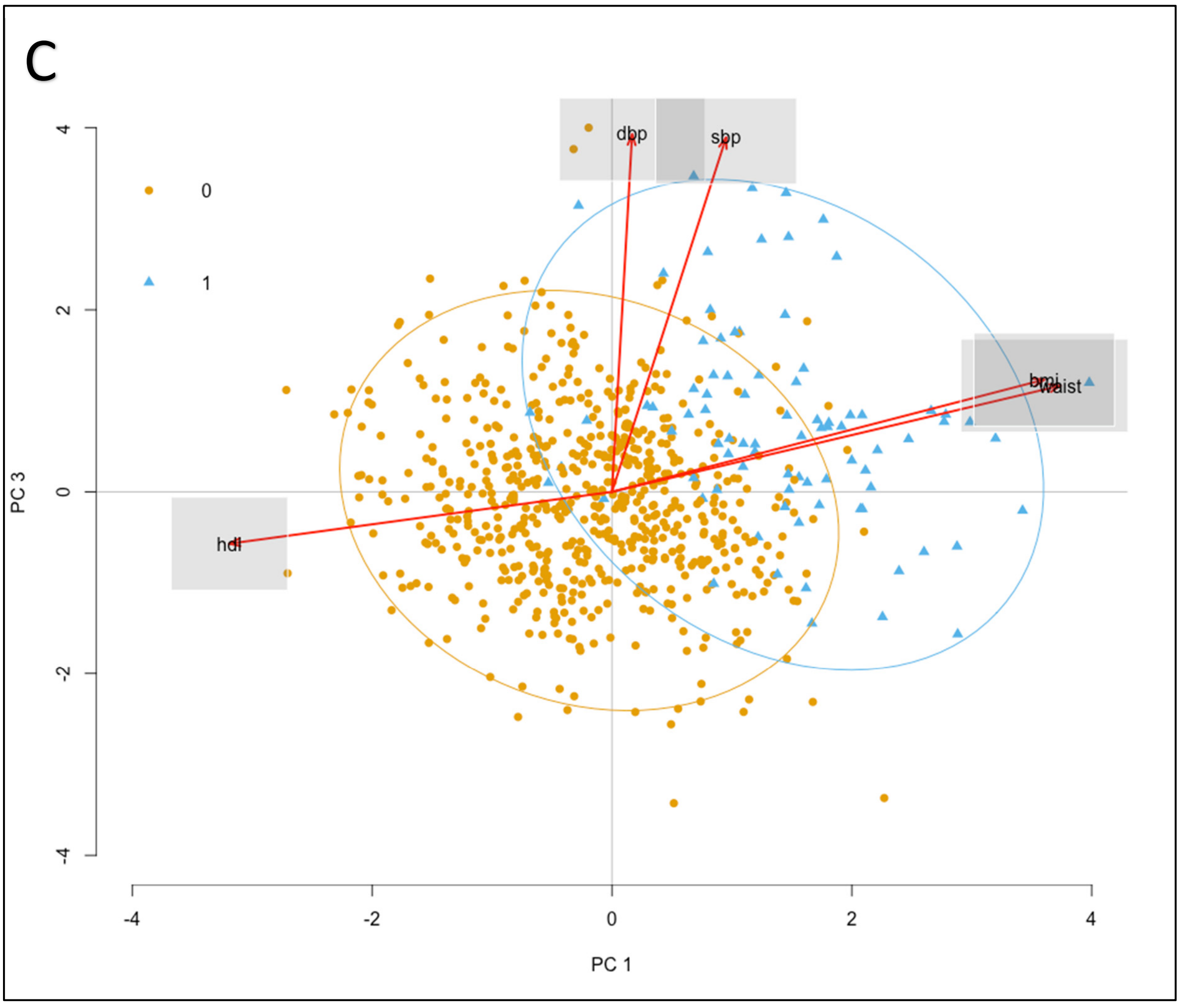
C
0
1
dbp
sbp
bmi
waist
hdl
PC 3
PC 1
4
2
0
-2
-4
-4
-2
0
2
4
